# Challenges in applying the EU AI act research exemptions to contemporary AI research

**DOI:** 10.1038/s41746-025-02263-0

**Published:** 2026-01-31

**Authors:** Janos Meszaros, Isabelle Huys, John P. A. Ioannidis

**Affiliations:** 1 Clinical Pharmacology and Pharmacotherapy, Department of Pharmaceutical and Pharmacological Sciences, KU Leuven, Leuven, KU Leuven Belgium; 2 Center for IT & IP law (CiTiP), Leuven, Belgium; 3 Meta-Research Innovation Center at Stanford (METRICS) and Departments of Medicine, of Epidemiology and Population Health, and Biomedical Data Science, Stanford University, Stanford, CA USA

**Keywords:** Ethics, Law, Drug development, Health policy, Health care, Medical research

## Abstract

The EU Artificial Intelligence Act (AI Act) is the world’s first comprehensive, cross-sectoral legal framework dedicated specifically to AI. It introduces a structured regulatory approach to ensure that AI systems are safe, transparent, and trustworthy. To foster innovation, it includes research exemptions that place certain AI systems - those under development or used solely for scientific research - outside of its scope and obligations. However, this paper argues that these exemptions rely on distinctions that may not fully capture the realities of contemporary AI research. These include the unclear divide between research and commercial activities, and between lab-based development and real-world testing. Through legal analysis and practical scenarios, we demonstrate how the blurred boundaries between academic and commercial interests, as well as between controlled research and real-world use, create regulatory uncertainty and open the door to potential misuse. The paper highlights the risks stemming from vague definitions and the lack of harmonized guidance. It ultimately calls for clearer guidance, stronger safeguards, and more realistic frameworks that reflect the complexities of modern AI research.

## Introduction

The emergence of artificial intelligence (AI) as a transformative technology has catalysed advancements across diverse sectors, from healthcare and education to finance and transportation^1,2^. However, these advancements bring complex ethical, legal, and societal challenges. In response, policymakers around the world have increasingly turned their attention to AI, with legislative discussions on the topic accelerating significantly in recent years, particularly in the United States (US) and the European Union (EU)^3,4^. The EU has enacted the AI Act^5^, a comprehensive legislative framework that governs the development and use of AI systems to ensure their safety, trustworthiness, and compliance with fundamental rights while fostering innovation^6^. This is particularly important given the well-documented concerns around reproducibility and the reliability of research findings, especially in fields like healthcare and medical research^7,8^, and the AI Act may have a significant impact on these fields^9–12^.

**Fig. 1:**
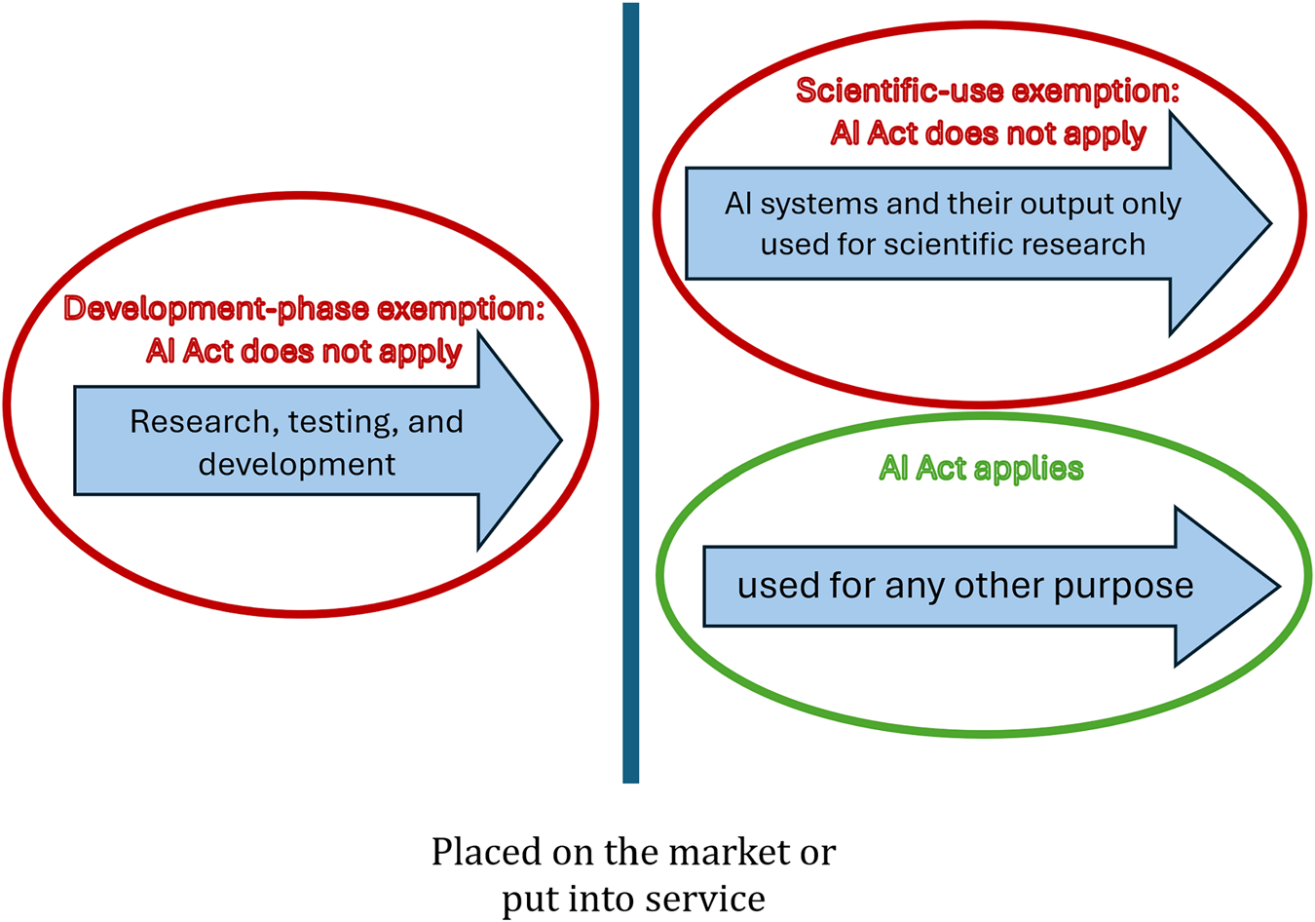
A Conceptual framework of the AI Act research exemptions based on development stage and intended use. This diagram illustrates the boundaries between activities excluded from the AI Act and those falling within its scope. The vertical line represents the critical regulatory threshold of being placed on the market or put into service, which generally triggers the Act’s obligations. Red ovals highlight the Development-phase exemption for research prior to market placement and the Scientific use exemption for systems used solely for research. The green oval signifies that the AI Act applies once a system is used for purposes other than exclusive scientific research.

Among its provisions, the Act includes two research exemptions, which aim to balance regulatory compliance with the promotion of scientific inquiry and innovation^13^. For the purposes of this paper, these will be referred to as the “development-phase exemption” and the “scientific-use exemption” (terms used here for clarity, as the AI Act itself does not formally label them this way). The development-phase exemption specifies that the AI Act does not apply to any research, testing, or development activities regarding AI systems or AI models before they are placed on the market or put into service. The scientific-use exemption clarifies that the Act does not apply to AI systems or models, including their output, specifically developed and put into service for the sole purpose of scientific research. The distinctions and overlap between these two exemptions are mapped in Fig. 1.

These provisions reflect the EU’s recognition of the value of scientific research to societal progress and its intent to foster innovation. However, as this paper argues, the boundaries these exemptions rely on are increasingly difficult to define in practice. In particular, the distinction between lab-based development and real-world use is often unclear in modern AI research, where systems may interact with live data or affect real-world outcomes even while nominally still in development. This blurred line raises critical questions about the scope and applicability of the AI Act, and whether the exemptions could be used to circumvent its obligations.

At the same time, the paper also considers how the evolving nature of research - where academic and commercial interests are often intertwined - challenges the assumption that scientific research can be neatly separated from other commercial activities. These dynamics create regulatory grey zones that could allow for strategic reclassification of AI systems to delay or avoid compliance.

This paper provides a detailed analysis of the AI Act’s research exemptions, examining their legal foundations, practical implications, and potential for misuse. It explores how the exemptions might be interpreted in ambiguous scenarios, particularly around real-world use, and assesses the risks of regulatory arbitrage, meaning situations where actors take advantage of gaps or ambiguities in the legal framework to avoid stricter requirements. By critically engaging with these issues, the paper highlights the challenge of designing AI regulation that both supports innovation and maintains clear, enforceable safeguards. This analysis is not only a contribution to the ongoing debate around the AI Act but also a broader reflection on the interplay between regulation and innovation in the age of AI. While our discussion focuses on the EU framework, the EU AI Act may set a precedent that other countries may take into account as they shape their own respective regulatory frameworks for AI^14,15^.

## Results

The AI Act’s scope is defined both in terms of the material aspects (the types of AI systems and activities it covers) and geographically (its applicability across different regions). The regulation is comprehensive, encompassing a broad range of actors involved in the development, deployment, and distribution of AI systems, often regardless of whether these actors are based within the EU or in a third country^16^. Therefore, the AI Act establishes a broad regulatory framework for AI systems, covering a wide range of actors involved in their development, deployment, and distribution, regardless of location. Despite its broad reach, the AI Act includes several exceptions. This paper focuses on two of them: the development-phase exemption and the scientific-use exemption, both of which are central to understanding how the Act treats research activities.

### Development-phase exemption

The development-phase exemption applies to all AI systems and models, before they are placed on the market or put into service: ‘*This Regulation does not apply to any research, testing or development activity regarding AI systems or AI models*^17^ *prior to their being placed on the market or put into service. Such activities shall be conducted in accordance with applicable Union law. Testing in real world conditions shall not be covered by that exclusion’*^18^.

The key elements are the following: (1) The AI system or model is still under development, in the ‘lab’; (2) Potentially, it can be a commercial product: the goal of the development can be to put it on the market; (3) Testing in real world conditions is not covered by the exemption.

Therefore, the main limitations for AI systems under the development-phase exemption are the following: (1) they cannot be placed on the market or put into service; (2) they can be tested in real world conditions only with the strict rules of the AI Act.

Putting into service means the first use for its intended purpose^19^. For instance, when a hospital begins using an AI system to diagnose patients. Placing on the market means offering an AI system to someone else in the EU, whether for payment or free of charge, so buyers can use it, whether or not they actually start using it right away. The AI Act also defines the process and requirements of ‘testing in real world conditions’^20,21^. This refers to the temporary use of an AI system outside the laboratory, with human participants who have given consent, and with the approval of relevant authorities. The goal is to collect data and verify conformity during the development phase, before the system is placed on the market or put into service^22^. In simpler terms, this amounts to putting into service in a controlled environment. For example, an AI system is used in a hospital for 6 months, with the consent of the patients and approval of relevant authorities. If the testing went well, it can be an important step in the development and verification of the system. Therefore, this testing is also a controlled process, based on the approval of authorities. However, the AI Act does not provide a definition for the concept of ‘real-world conditions’ itself. Instead, its meaning can only be inferred from the Act’s provisions related to the process and requirements. This interpretative gap is important, because it determines when research and development activities are deemed to leave the lab and fall under the AI Act.

From these limitations, it is clear that an AI system under the development-phase exemption cannot be put into service and therefore cannot influence real-world decisions about individuals. For instance, a physician cannot rely on the system’s predictions or recommendations when treating a patient. It would be only possible if the AI Act applies. But what about the reverse scenario? Suppose an AI system is silently present in a hospital room, capturing conversations, diagnoses, and patient data, and transmitting them back to a laboratory without generating any outputs at the point of care. Would this still qualify for the development-phase exemption?

Whether silent-mode deployment qualifies as ‘testing in real world conditions’ depends on the system’s intended purpose. The AI Act defines ‘testing in real world conditions’ as the temporary testing of an AI system for its intended purpose outside the laboratory. If the system is designed for diagnosis or treatment support, then running it in silent mode in a hospital - even without showing outputs to clinicians - may still amount to testing it for that purpose. In such cases, the activity might fall under real-world testing rather than the development-phase exemption and thus trigger the requirements of the AI Act, including prior approval, informed consent, oversight, and time limits. By contrast, if the system’s hospital presence is limited to functions unrelated to its clinical purpose, such as hardware checks, data-transfer pipelines, or robustness to noise, it may still be regarded as remaining within the development phase.

This creates a critical ambiguity for researchers: while lab-based development is excluded from the Regulation, once the AI system is tested in a real-world environment—even if still under development—it falls within the scope of the Act. While the Act sets rigorous and comprehensive standards to ensure the safe and ethical deployment of AI, a key area of concern is the distinction between lab testing and real-world testing, where some actors may try to exploit regulatory grey areas to conduct de facto real-world testing under the guise of research exemption. To illustrate these risks, Table 1 outlines several hypothetical circumvention methods.

**Table 1:** ypothetical circumvention methods for using real-world datad

Circumvention Method	Description	Issues
Offshore Cloud Deployment	Testing the AI system in jurisdictions outside the EU. Hypothetical Implementation: A company in Belgium collects data to train a model. Then it hires a company in India to test the model on patients in India.	Regulatory bodies may have difficulty enforcing compliance on non-EU infrastructure.
Synthetic Environment with Live Overlays	Using a synthetic or simulated environment that is periodically updated with small amounts of live data to mimic real-world conditions. Hypothetical Implementation: Developers claim the system is being tested in a fully controlled simulation while, in reality, live data injections subtly alter the environment to replicate live conditions.	Blurs the line between controlled simulation and real-world deployment
Model-Splitting Across Systems	Developers divide a multi-model AI into several systems. One system is approved for real-world testing, while the others stay in the lab under the development-phase exemption. Hypothetical Implementation: A diagnostic platform is split into three systems: one image-preprocessing model is submitted for real-world testing, while two diagnostic models are kept in the lab.	Allows undeclared models to exploit real-world conditions indirectly, creating a regulatory gap between declared and hidden testing.
Silent On-Site Data Capture	An AI system is placed in a real-world setting (e.g., hospital) in silent mode. It only records and sends live data back to the lab, without showing outputs at the hospital. Hypothetical Implementation: A device in a hospital room streams patient–doctor interactions to the developer’s lab, where predictions are tested.	Although outputs stay in the lab, the system still operates outside of it.

The scenarios presented in this table are purely hypothetical and are provided solely for academic and research purposes, to help identify and address potential regulatory gaps. It is important to note that if any of these activities affect individuals within the European Union, the provisions of the EU AI Act will apply. Consequently, employing these circumvention methods in practice would likely constitute a violation of the AI Act and could also infringe upon other applicable regulations, such as the EU General Data Protection Regulation (GDPR). All entities are expected to fully comply with the relevant legal frameworks to ensure the ethical and lawful deployment of AI systems.

The examples outlined in the table illustrate how some actors might attempt to circumvent the AI Act’s strict regulatory requirements by exploiting ambiguities in the distinction between lab-based research and real-world testing. However, such strategies are unlikely to succeed under Article 2, which establishes that the AI Act applies to any system that impacts individuals within the EU, regardless of where the system or its provider is located. Furthermore, Recital 22 reinforces this broad scope, clarifying that the Act applies even when an AI system is hosted outside the EU but still produces outputs affecting EU users^23^. These insights highlight the need for strong enforcement mechanisms and clear regulatory guidance to ensure that AI systems - regardless of how and where they are tested or deployed - undergo proper risk assessment and accountability measures. By addressing these potential circumvention methods proactively, policymakers and regulators can strengthen the AI Act’s effectiveness and prevent attempts to undermine its intended protections.

### Scientific-use exemption

The scientific-use exemption is about the AI systems and models, that were made only for research purposes: ‘This Regulation does not apply to AI systems or AI models, including their output, specifically developed and put into service for the sole purpose of scientific research and development.’^24^

Here the key elements are the following: (1) The AI system or model is already finished; (2) It has been put into service; (3) It was developed for research purposes; (4) It was put into service for research purposes.

This exemption raises several questions and unclear situations, which we are addressing in this section. If an AI system is developed and put into service or placed on the market solely for research, it is exempted from the AI Act. If either condition is not met, the exemption does not apply. However, interpreting “sole purpose” is problematic, since goals and intentions can change over time, and proving original intent can be difficult. This creates room for potentially questionable practices, such as presenting a commercially driven system as research to avoid regulation. These ambiguities lead to four possible scenarios, demonstrated in Table 2.

**Table 2:** The interpretation of research exemption in the AI Act

Developed	Put into service/Placed on the market	Does the research-use exemption apply?
✔ For the sole purpose of scientific research	✔ For the sole purpose of scientific research	✔ Yes.
✔ For the sole purpose of scientific research	Ø NOT For the sole purpose of scientific research	Ø No.
Ø NOT for the sole purpose of scientific research	✔ For the sole purpose of scientific research	Unclear: by definition, the exemption should not apply, but it would not be logical. E.g. if an AI system was developed for screening patients, but the developers changed their mind, and they deployed it only for research on medical images.
Ø NOT for the sole purpose of scientific research	Ø NOT for the sole purpose of scientific research	Ø No.

As the table shows, the text of the AI Act creates gaps and maybe even counterintuitive outcomes. Recital 25 of the AI Act provides some clarification: it states that, apart from AI systems developed and used solely for scientific research, any other AI system that can be used in research and development activities remains subject to the Regulation^25^. For instance, if a hospital purchases a commercial AI screening tool but decides to use it only for research, the exemption does not apply. While recitals are not legally binding, they are important in guiding the interpretation of the binding provisions. Table 3 below provides practical examples of AI use cases and evaluates whether they could potentially qualify for the exemption. AI systems used strictly for research in laboratory environments, or academic studies generally remain exempt. However, once an AI system is deployed in practical applications, such as diagnosis in healthcare, it falls outside the exemption and becomes subject to AI Act regulations. The examples illustrate how AI systems can transition from scientific research to real-world deployment, marking the point where compliance obligations begin.

**Table 3:** Examples for the scientific-use exemption

Scenario	Is It Exempt?	Why?
A university has developed and put into service an AI system that analyses cancer images in a laboratory environment only for scientific research, without using it for patient diagnosis.	Exempt ✔	The AI system was developed and put into service only for scientific research.
A hospital’s research unit has developed an AI system for diagnosing lung cancer and has now implemented it in routine patient care.	Not exempt Ø	The AI system was developed and put into service beyond research (real medical use).
A company has developed an AI system for diagnosing lung cancer. A university bought the system, but implemented only for doing research (no diagnosis, no patient care).	Not Exempt Ø	The AI system was not developed solely for scientific research. Just because the university uses it only for research, it does not qualify for the exemption.
An AI system was developed to detect melanoma from patients’ skin lesion images at a hospital. However, the developers changed their mind during the development-phase, and they continued to develop it only for research. Also, after the system was ready, it was put into service only for research purposes.	Unclear **?**	It is unclear whether the ‘sole purpose’ should apply for the whole research process.

The examples in Table 3 illustrate the complexity of applying the research exemption under the AI Act. While some scenarios clearly fall within the exemption, others expose grey areas where the boundary between research and real-world deployment is unclear. This lack of clarity raises concerns about regulatory arbitrage, where entities may classify AI applications as “research” to bypass compliance requirements, even when they aim or hope to have tangible real-world impact. In many cases, early developers may even have in all honesty little ability to judge the commercial and real-life potential of what they work on as a research enterprise. This ambiguity is further compounded by the absence of a harmonized definition of ‘scientific research’ in EU law^26,27^. Similar difficulties arise under the EU General Data Protection Regulation^28^ and the EU Copyright Directive^29^, both of which include research-related exemptions but leave the concept of “scientific research” only loosely defined.

The AI Act appears to assume that research exists as a distinct and isolated activity, separate from commercial or practical applications. However, in reality, modern research is highly interconnected, involving collaborations across academic institutions, public bodies, and private enterprises^30–32^. Some funding agencies even require (as a pre-condition for funding) the partnership between non-profit research institutions with private companies, even for research that has no clear translational potential to real-life applications when it starts. As research ecosystems become more multidisciplinary and intertwined with commercial interests, the distinction between scientific inquiry and research serving strategic or commercial purposes becomes increasingly blurred. Even with full honesty and no hidden agendas, many investigators and other AI stakeholders may have difficulty to tell whether what they do is exclusively curiosity-driven research or may have applications. This regulatory gap not only creates legal uncertainty but also opens pathways for exploitation of exemptions, allowing AI projects to be labelled as research while still influencing real-world decisions.

### Challenges and issues

The EU AI Act assumes a clear-cut distinction between research and commercialization, but in reality, modern research often operates on a spectrum rather than a binary divide. Many research projects are conducted with the potential for later commercialization, particularly in public-private partnerships or university-industry collaborations^33,34^. Several questions arise. For instance, if an AI model is initially developed exclusively for research but later deemed commercially valuable outside research, does it retroactively lose the research exemption status? Or it can be never used outside research?

Similar tensions arise in publicly funded research. Funding bodies often impose knowledge-transfer obligations requiring investigators to share findings with broader communities, including industry partners^35,36^. Universities and research institutions often enter into partnerships with companies that may eventually use research outputs commercially^37^. Public funders may even require such partnerships to be present as a pre-condition for funding. The exemption’s strict language could hinder such collaborations or require additional compliance measures.

The situation is equally complex for non-profit and humanitarian AI initiatives. Many AI research projects, particularly those aimed at social benefit (e.g., climate modelling, disease prediction, disaster response)^38^, are designed to be freely available to public institutions, governments, non-profit organizations, and the general public. If providing such AI models to external stakeholders constitutes real-world use, it could force researchers to comply with the full AI Act requirements even when there is clearly no commercial intent. Commercial for-profit stakeholders (mostly from outside the EU) may then occupy that space that could have otherwise been covered by not-for-profit products.

Ambiguity in the research exemption could also enable regulatory arbitrage. Companies might attempt to exploit the research exemption by initially developing AI models under the guise of scientific research before transitioning them to commercial applications. This could create a regulatory grey area where companies avoid early compliance costs. Conversely, overly rigid enforcement of the restriction could lead to the opposite problem: legitimate research efforts being unfairly classified as commercial, forcing researchers to navigate regulatory hurdles that could slow down innovation; or even offering disincentives to doing this research; or incentives to do the research and development in countries with less strict regulations

Finally, the notion of “real-world testing” remains poorly defined, which is a fundamental component of AI research, often requiring deployment in limited external environments^39–41^. The AI Act’s development-phase exemption excludes ‘testing in real world conditions’, but does not define what constitutes “real-world conditions”. This creates challenges for researchers working with live or real-time data in controlled settings. Some actors may exploit this grey area to conduct near-deployment testing while claiming exemption under the guise of research. Although Article 2 and Recital 22 aim to close jurisdictional loopholes, the blurred boundary between lab-based testing and real-world impact calls for clearer definitions.

## Discussion

To ensure the AI Act’s research exemptions support genuine scientific progress without enabling regulatory loopholes or misuse, several steps should be considered.

First, the definition and scope of the research exemptions require greater clarification. Establishing clearer and more robust definitions of scientific research and real-world conditions could help prevent unintended regulatory gaps. This could include criteria for distinguishing between activities that qualify for the exemptions and those that do not, as well as a process for assessing when an AI system transitions from research into real-world use. For the development-phase exemption, particular attention is needed regarding silent-mode deployments in real-world settings, when the AI system only collects live data, without providing predictions, recommendations or decisions. Even when outputs are not shown to clinicians, such practices may still be regarded as real-world testing and therefore fall outside the exemption, since the system is tested for its intended purpose. For the scientific-use exemption, clarification would also be useful on the requirement that systems be both developed and put into service solely for scientific research. While the public or private nature of an organisation may serve as an indication, the decisive factor is whether the activity is carried out exclusively for scientific research. In practice, research in public organisations is often more transparent, which may make it easier to demonstrate compliance with this requirement.

Second, the EU should facilitate responsible knowledge transfer between academic and commercial entities while ensuring that research exemptions are not misused. Many publicly funded research initiatives have obligations to share findings with industry stakeholders, yet the current wording of the exemption does not sufficiently account for these collaborations. A structured approach is needed to allow research findings to be transferred responsibly without undermining the Act’s regulatory objectives. This could include transparency requirements for research projects involving private sector partnerships, ensuring that exemptions do not provide a backdoor for unregulated commercialization.

Third, practical implementation guidelines should be developed for the AI Act to assist researchers, regulators, and industry stakeholders in applying the AI Act’s research exemptions consistently. A set of best practices should be published, outlining how research institutions and companies can navigate the exemptions while remaining compliant with broader AI regulations. This should be complemented by ongoing dialogue between policymakers, the research community, and industry representatives to refine the exemptions based on real-world challenges and emerging developments in AI research. Given that the capacity and possibilities of AI change rapidly, these practical guidelines need to be re-evaluated to be kept relevant and up to date.

By addressing these concerns, the AI Act can strike a balance between fostering scientific progress and maintaining regulatory safeguards. This approach could ensure that AI research continues to drive innovation while upholding ethical, legal, and societal responsibilities. Furthermore, the global landscape should be considered, as the regulatory environment regarding AI may change over time in different countries. This may create advantages and disadvantages for research stakeholders and for those who seek to develop products. Such imbalances may result in shifting research and development away from some countries towards others that have more permissive regulatory settings or even no regulatory oversight at all. High-risk products developed in such settings may then feed into more global markets, creating problems that could have been anticipated and managed earlier otherwise.

The AI Act’s research exemptions are designed to foster innovation, yet they rest on an outdated assumption that scientific research and real-world application can be clearly separated. This paper has shown that the boundary between lab-based research and real-world testing is increasingly blurred, especially in AI development, where live data and deployment-like conditions are often essential. Without clearer definitions and safeguards, these exemptions risk becoming loopholes, enabling regulatory avoidance under the guise of research. Strengthening guidance and oversight is crucial to ensure the Act supports responsible innovation while maintaining public trust and legal clarity.

## Methods

This study uses a doctrinal legal research approach based on the analysis of publicly available legal and regulatory sources. Primary materials include the EU Artificial Intelligence Act, related EU legislation, and European Commission guidance. Secondary sources, including academic literature, policy papers, and technical reports, were identified through targeted database searches and reference screening. The analysis applies legal interpretation and conceptual evaluation of the EU AI Act research exemptions, supplemented by scenario-based reasoning to illustrate practical implications and areas of ambiguity. No empirical data or experimental results were generated or analysed.

## Data Availability

This work does not involve the generation or analysis of datasets. All legal, regulatory, and academic sources necessary to interpret and verify the analysis are publicly available and cited within the manuscript.
